# What is the Minimal Surgery for Papillary Thyroid Carcinoma?

**DOI:** 10.5041/RMMJ.10232

**Published:** 2016-01-28

**Authors:** Eran Fridman, Ziv Gil

**Affiliations:** Department of Otolaryngology Head and Neck Surgery, The Head and Neck Center, the Clinical Research Institute, Rambam Health Care Campus, Rappaport Institute of Research and Medicine, The Technion-Israel Institute of Technology, Haifa, Israel

**Keywords:** Hemithyroidectomy, lobectomy, papillary, thyroid

## Abstract

Although thyroid surgery for treatment of papillary thyroid carcinoma (PTC) has been practiced for more than 100 years, there is still controversy regarding the minimal surgery needed for cure. The main reason for this controversy is lack of prospective randomized trials. The data accumulated in the last four decades indicate that hemithyroidectomy can be sufficient and safely practiced in low-risk patients with PTC. Patients <45 years of age with a single tumor less than 2 cm, with no lymphatic spread, and in the absence of other risk factors, can be equally managed by hemithyroidectomy or total thyroidectomy. A slight increase in the risk of vocal cord paralysis and hypocalcemia after total thyroidectomy suggests that hemithyroidectomy is appropriate for the management of patients with stage T1 disease. Any choice regarding the extent of surgery should be made with the patient and his family and in a multidisciplinary setup, which has been shown to improve decision-making procedures before the operation and during follow-up.

## INTRODUCTION

Thyroid cancer incidence has tripled over the last three decades, from 4.9 to 14.3 per 100,000 people. This is almost exclusively due to an increase in the incidence of early-stage papillary thyroid carcinoma (PTC), from 3.4 to 12.5 per 100,000 people.^1^ Nevertheless, the increase in diagnosis of early-stage PTC did not decrease the mortality from this disease, which has remained unchanged during this period.^1^,^2^ The increase in surgeries for resection of <2 cm PTC is considered to be mainly due to the accessibility of high-resolution ultrasound and the increase in use of routine imaging modalities of the neck for other diseases.^2^–^4^ In agreement with the utility of imaging modalities, the number of more advanced PTC diagnosed has also increased.^5^ Papillary thyroid carcinoma has generally a better outcome compared to other thyroid cancer types, with a 5-year overall survival (OS) of 97%–99%.^6^ Nevertheless, there is a subset of patients that face a worse prognosis, and it is important to recognize and distinguish these patients in order to provide adequate treatment that can improve outcome.

A number of staging systems have been suggested in order to distinguish between low- and high-risk patients. The Mayo Clinic offered the MACIS system (metastasis, age, completeness of resection, invasion, and size),^7^ while the Memorial Sloan–Kettering Cancer Center divided patients to low-, intermediate-, and high-risk patients using age, the presence of distant metastasis, tumor size, and histology. Both systems found excellent survival rates for low-risk patients, which represent >80% of the patients, with a 5-year OS of 100% and a 20-year OS of 99%.^8^–^10^ In accordance with these systems, the American Joint Committee on Cancer (AJCC) published a TNM staging system which takes into account several risk factors including patient age, tumor size, histopathological type, status of lymph nodes, and presence of distant metastasis.^11^

In all of these staging systems, the cutoff size for T1 classification is tumor size <2 cm. This distinction emerged since the survival rate for tumors <2 cm is ∼99%.^10^

Since the beginning of the twentieth century, surgery has remained the main modality of treatment for PTC.^12^ Although thyroid surgery has been practiced for more than 100 years, there is still controversy regarding the minimal surgery for treatment of PTC. In this paper we will describe the factors which influence the decision regarding the extent of thyroid surgery in patients with low-risk PTC.

## THE EXTENT OF THYROID SURGERY

The goals of treatment of patients with low-risk PTC are: to improve overall survival, to reduce the rate of recurrence, and to minimize complications. The main reason for the controversy of the minimal surgery for PTC is the lack of prospective randomized studies. Since the biology of this slow-growing disease will require large cohorts of patients and very long follow-up, such studies are not expected any time soon.

Although the American Thyroid Association and the National Cancer Comprehensive Network recommended that patients with <1 cm PTC should undergo hemithyroidectomy in the absence of contralateral nodes, lymphatic spread, and distant metastases, there are still clinicians that would perform total thyroidectomy on low-risk patients. Another disagreement is regarding the surgical treatment of low-risk patients with tumors sized 1–4 cm.

Since 1940 there has been a sharp decline in the number of patients undergoing hemithyroidectomy. However, since 1985, the pendulum has shifted back to hemithyroidectomy as the modality of treatment of low-risk PTC.^13^ There are a number of considerations in favor of total thyroidectomy: it enables the use of radioactive iodine (RAI) after surgery, patients can be treated with thyroid-stimulating hormone (TSH) suppression, and follow-up can be easily and inexpensively performed using thyroglobulin measurement. It also minimizes the risk of leaving tumor tissue behind, in cases of multifocal and bilateral disease.

Total thyroidectomy, which requires the removal of all thyroid tissue, puts at higher risk recurrent and superior laryngeal nerve, and the parathyroid glands and their vascular supply, compared to hemithyroidectomy, which involves removal of one lobe only.^14^ As a result, the complication rate is significantly higher in the case of total thyroidectomy. Kandil et al. conducted a meta-analysis, examining 50,445 patients who underwent thyroid surgery (30% hemithyroidectomy versus 70% total thyroidectomy). They reported a higher relative risk (RR) for all complications in the total thyroidectomy group.^15^ Specifically, the RR was 10.67 for temporary hypocalcemia, 3.17 for permanent hypocalcemia, 1.69 and 1.89 for temporary and permanent injury to the recurrent laryngeal nerve, and 2.58 for hemorrhage. Another potential disadvantage of total thyroidectomy is the need for lifelong thyroid hormone replacement. The need for long thyroid hormone replacement in patients undergoing hemithyroidectomy was estimated in a recent meta-analysis of 4,899 patients.^16^ The weighted pooled incidence of hypothyroidism after hemithyroidectomy was 21% (95% CI 17–25), but only 4% (95% CI 2–8) had symptoms as a result.

The prognosis of patients with PTC <1 cm (also known as microcarcinoma) is excellent, and most guidelines recommend hemithyroidectomy for these tumors in the absence of the following risk factors: lymphatic spread, distant metastases, past radiation, macroscopic multifocal disease, or extra-capsular extension.^17^ Only the Latin American Thyroid Society recommends total thyroidectomy for all PTC regardless of size.^18^ Lately several authors have been arguing for an even more conservative approach towards PTC. This approach was first suggested by Ito et al. who followed 340 patients with PTC <1 cm. Of these, 109 (32%) underwent surgery during the observation period with no effect on nodal metastasis.^19^ The authors concluded that surveillance instead of surgery is an accepted modus operandi. Similarly Castro at al. from the Mayo Clinic support this view.^20^ In a report of 1,465 from two different groups (who were followed using the active surveillance approach which included ultrasound at 6 months and then once a year), during an average of 75 months of follow-up only 14% had surgery, and there was no disease-specific mortality.^21^,^22^

## THE CONTROVERSY OVER THE BILIMORIA STUDY

The largest study that showed survival benefit for patients undergoing total thyroidectomy was by Bilimoria et al.^23^ They used the National Cancer Data Base (NCDB) and conducted analysis of 52,173 patients recruited from multiple centers in the United States. The conclusion of the authors was that total thyroidectomy for PTC > 1 cm is associated with better outcome, in terms of recurrence and OS. The data influenced future guidelines of several societies; however, re-evaluation of the data from this study raised significant concerns regarding the validity of their conclusions. First, when examining the patient characteristics, one can see that 761 (10.5%) of patients that had hemithyroidectomy had nodal disease prior to surgery. This means the extent of the initial surgery was inadequate. Second, 723 (18.4%) of the patients that were reported to undergo hemithyroidectomy also received adjuvant RAI. This piece of data questions the validity of the database which the study is based on. It also suggests the initial surgery was inadequate. Third, the overall difference between hemithyroidectomy and total thyroidectomy when including all comers, in terms of 20 year survival, is ∼1%, also when including patients with >4 cm tumors. This finding suggests that statistical differences which are frequently found in large cohorts have minimal clinical significance. Shah^24^ and Yu et al.^25^ have also raised some limitations and opt for careful drawing of conclusions and making of recommendations based on Bilimoria’s data. Adam et al.^6^ used the same database (NCDB), analyzing 61,775 patients, with tumor size of 1–4 cm, who underwent total or hemithyroidectomy. They found no difference in OS between the two groups. Table 1 lists articles demonstrating that hemithyroidectomy is an adequate surgery compared to total thyroidectomy in terms of measured outcome.

**Table 1.** Data Showing that Hemithyroidectomy Is Equal to Total Thyroidectomy in Terms of Measured Outcome.

**Author**	**Year**	***n***	**Database/Institute**	**Conclusion**
Shaha AR, et al.^26^	1997	1,038	Memorial Sloan–Kettering Cancer Center, New York, USA	No statistical difference in local recurrence rate, overall failure rate
Wanbeo H, et al.^27^	1998	347	Brown University, Providence, Rhode Island, USA	Total is equal to hemithyroidectomy in high-risk patients with differentiated thyroid cancer (follicular histology, vascular invasion, or extra-capsular extension)
Hundahl SA, et al.^28^	1998	53,856	NCDB	Extent of surgery has no impact on survival in any subgroup of papillary or follicular carcinoma
Haigh PI, et al.^29^	2005	5,432	SEER	No difference in outcome between groups for both low- and high-risk patients
Vorburger SA, et al.^30^	2009	2,867	Inselspital, Bern, Switzerland	No difference in outcome between groups
Mendelsohn AH, et al.^31^	2010	22,274	SEER	No differences in overall or disease-specific survival
Barney BM, et al.^32^	2011	23,605	SEER	No difference in outcome between groups
Nixon IJ, et al.^33^	2012	889	Memorial Sloan–Kettering Cancer Center, New York, USA	No difference in local recurrence or regional recurrence between groups
Ebina A, et al.^34^	2014	1,187	Cancer Institute Hospital, Tokyo, Japan	No difference in outcome between groups
Nilubol and Kebebew^35^	2014	61,523	SEER	No difference in outcome between groups
Adam MA, et al.^6^	2014	61,775	NCDB	No difference in outcome between groups

## THE ROLE OF POSTOPERATIVE RAI TREATMENT

The follow-up of patients with low-risk PTC includes periodical physical examination, ultrasound, and monitoring of TSH, anti-thyroglobulin Ab, and thyroglobulin levels. The last-mentioned is significant only after total thyroid resection and RAI therapy. Postoperative RAI treatment can improve loco-regional control in high-risk patients; however, it has no impact on outcome of patients with stage I disease. The use of RAI is also used for ablation of residual thyroid tissue and for imaging for metastatic or recurrent disease. Podnos et al. have reported that only high-risk patients (age >45, tumor size >2 cm, presence of lymph node metastases and distant metastases) benefit from postoperative RAI.^36^ In a review on the role of postoperative RAI, Patel and Goldfarb have summarized that for the low-risk patients there is no outcome benefit using RAI.^37^ Mendelsohn et al. have found better OS for patients treated with RAI but decreased disease-specific survival (DSS).^31^ In the recently published 2015 American Thyroid Association Management Guidelines for Adult Patients with Thyroid Nodules and Differentiated Thyroid Cancer, the authors conclude that there is no benefit of RAI treatment in patients with low-risk PTC.^12^ One should also bear in mind the frequent adverse effects of RAI which involves dysgeusia, salivary gland inflammation,^38^ and an increase in the risk of salivary gland malignancies and leukemia.^39^

## MULTIFOCAL PAPILLARY CARCINOMA

Multifocal disease is two or more distinct PTC lesions found on a specimen. It can arise from the same tumor (same clone) or a second primary PTC.^40^ Those who side for total thyroidectomy frequently argue that PTC is a multifocal disease irrespective of size and therefore every patient with papillary cancer should undergo total thyroid ablation. Lucchini et al. have reviewed the records of 538 patients from a single institute that had undergone total thyroidectomy. They found a prevalence of 43% multifocal disease regardless of tumor size.^41^ Kim et al. studied 2,039 patients with PTC and concluded that multifocal disease has a prognostic value only for tumors >1 cm.^42^ However, they found no benefit of total thyroidectomy in these cases. Other studies also reported conflicting results on the risk of contralateral microcarcinoma in PTC <1 cm.^43^,^44^ Nevertheless, all the aforementioned studies found no difference in outcome between the extent of the resection and outcome.

## CONCLUSIONS

The data on PTC accumulated in the last four decades indicate that hemithyroidectomy can be safely practiced in low-risk patients with PTC. Patients with a single nodule less than 2 cm, age <45 years, without nodal metastases, and with no other risk factors can benefit from either total or hemithyroidectomy. The slight increase in the risk of vocal cord paralysis and hypocalcemia after total thyroidectomy suggests that hemithyroidectomy is appropriate for the management of patients with the stage T1N0M0 classification. Any choice regarding the extent of surgery should be made with the patient and his family, in a multidisciplinary setup, which improves decision-making before the operation and the management during the long-term follow-up needed.

## Abbreviations:

DSS: disease-specific survival
NCDB: National Cancer Data Base
OS: overall survival
PTC: papillary thyroid carcinoma
RAI: radioactive iodine.
